# Optimising mopane worm (*Gonimbrasia belina*) processing for improved nutritional and microbial quality

**DOI:** 10.3920/JIFF2022.0046

**Published:** 2023-04-07

**Authors:** F. Matiza Ruzengwe, F.A. Manditsera, O.N. Madimutsa, L. Macheka, G. Kembo, A. Fiore, M. Ledbetter, J. Mubaiwa

**Affiliations:** 1 Department of Food Science and Technology, Chinhoyi University of Technology, P. Bag 7724, Chinhoyi, Zimbabwe; 2 Centre for Innovation and Technology Transfer, Marondera University of Agricultural Sciences and Technology, P.O Box 35, Marondera, Zimbabwe; 3 Food and Nutrition Council of Zimbabwe, 1574 Alpes Road, Hatcliffe, Harare, Zimbabwe; 4 School of Applied Science, Division of Engineering and Food Science, Abertay University, Bell St, Dundee DD1 1HG, United Kingdom

**Keywords:** boiling time, drying temperature, optimisation, Mopane worms (*Gonimbrasia belina*), nutritional quality, microbial quality

## Abstract

Mopane worms (*Gonimbrasia belina*) is an important source of food and contribute to the nutrition of people who consume them. However, the traditional processing methods may have detrimental effects on the nutritional value and should also guarantee microbial quality. In this study, the nutritional composition and microbial quality of mopane worms processed under different boiling time (0-20 min) and drying temperature (40-60 °C) conditions were investigated and optimised using response surface methodology. An increase in the boiling time at the lowest drying temperature resulted in an increase in protein content and reduction in coliform counts. The optimum conditions of boiling for 20 min and drying at 40 °C resulted in mopane worms with a protein content of 49.4% DW and coliform counts <1.5 log cfu/g. In addition, high concentrations of crude fibre (13.6% DW) and fat (20.2% DW), as well as Fe (19.0 mg/100 g) and Zn (17.9 mg/100 g) were also recorded. A decrease in the total bacterial count, *Escherichia coli* and yeasts and moulds at the boiling time ≥20 min irrespective of the drying temperature suggested that exposure to heat reduced the microbial growth and contamination. Reduction of the mopane worms’ moisture content (<7%) due to drying further slowed down the rate of microbial growth. The optimal processing conditions (boiling for 20 min and drying at 40 °C) are recommended for pretreatment of mopane worms prior to further processing into various products.

## 1 Introduction

The need to diversify food sources that contain sustainable proteins has contributed to increased interest in edible insects for both direct human consumption and for development of novel products. The need for the production of sustainable proteins for human consumption is expected to continue increasing due to rise in world population projected to reach 9.8 billion by the year 2050 (UN, 2017). Edible insects offer an alternative and sustainable source of protein and minerals (Matiza Ruzengwe *et al*., 2022; Van Huis, 2013). In addition to this, edible-insects consumption contributes to addressing different forms of global malnutrition (Hlongwane *et al*., 2021).

Mopane worms (*Gonimbrasia belina*) is one of the commonly consumed wild insects in Southern Africa (Hlongwane *et al*., 2021; Mujuru *et al*., 2014). The insects have a high protein content ranging between 52-61% DW (Hlongwane *et al*., 2021; Madibela *et al*., 2007; Madibela *et al*., 2009; Payne *et al*., 2016,), zinc (10.8-13.3 mg/100 g DW) and iron (9.7- 29 mg/100 g DW) (Hlongwane *et al*., 2021). However, reported challenges associated with the wild harvesting of edible insects, include their seasonal availability (Manditsera *et al*., 2019), sustainability concerns, pathogens risk and high perishability (Fombong *et al*.,2017; Garofalo *et al*., 2017; Grabowski and Klein, 2017a,b; Rumpold and Schluter, 2013). To reduce some of these risks, mopane worms are subjected to primary processing after harvesting i.e. either boiling, or hot-ambers roasting followed by drying. Unfortunately, these traditional methods are usually uncontrolled, and this has negative effects on the nutritional content and safety of the edible insect (Kinyuru *et al*., 2010).

Several studies have reported on the effect of processing method on the edible insect nutritional properties (Lautenschläger *et al*., 2017; Madibela *et al*., 2007, 2009; Manditsera *et al*., 2019; Megido *et al*., 2018). However, it is difficult to generalise for all insects as these properties are also dependent on the insect species and geographical region (Mutungi *et al*., 2019). Previous studies have reported that boiling mopane worms for 60 min significantly reduced its protein content (Madibela *et al*., 2007). Conversely, the protein content of boiling for 30 min and a combination of boiling for 30 min and drying edible caterpillars *(Imbrasia epimethea*) another member of lepidoptera was not significantly changed (Lautenschläger *et al*., 2017). Manditsera *et al*. (2019), investigated the effect of boiling (30 min, 60 min and twice boiling for 30 min) of beetles (*Eulepida mashona*) and cricket (*Henicus whellani*). A significant decrease on the protein content after boiling the crickets for 30 min and the beetles for 60 min and twice for 30 min was reported in the study (Manditsera *et al*., 2019). No effect was recorded for the iron and zinc content for both insects. Thus, the nutrient composition of edible insects varies with processing times (Matiza Ruzengwe *et al*., 2022), necessitating process optimisation to maximise the nutritional quality.

Heating has an impact on the microbial quality of food by inactivating some of the pathogenic microorganisms (Wynants *et al*., 2018). Dry heat treatment was reported to be less effective as compared to a combination of 5 min blanching and roasting in reducing the microbial load in mealworms (*Tenebrio molitor*), house crickets (*Acheta domesticus*) and large crickets (*Branchytrupus* sp.) (Klunder *et al*., 2012). Mujuru *et al*. (2014) evaluated the microbiological quality of mopane worms processed under various traditional practices (ash drying, drum roasting, boiling in salt water followed by solar drying and boiling in salt water and subsequent open pan roasting). The results of the study highlighted boiling in salt water and subsequent open pan roasting as the most effective process in reducing the microbial load. Coliforms, *Staphylococcus aureus*, *Salmonella* spp. and *Escherichia coli* were eliminated by boiling in salt water and subsequent roasting. Microorganisms in mopane worms can be found on the skin and gut contents (Braide, 2012), highlighting contamination could be well before human handling and manipulation (Belluco *et al*., 2013). Coliforms and *E. coli* are indicator microorganisms that have been reported to be present in mopane worms (Mujuru *et al*., 2014). Therefore, the aim of the study was to investigate the effect of boiling time and drying temperature on the nutritional composition and microbial load of mopane worms. The influence of processing conditions (boiling time and drying temperature) was optimised for the protein content and the coliform counts in the processed mopane worms using response surface methodology, precisely central composite design. Since the presence of coliforms signifies danger to public health (Mujuru *et al*., 2014) it is important to investigate them in mopane worms after processing. Focus on protein content optimisation is due to the need for alternative sustainable protein sources that can help meet the anticipated rise in world population.

## 2 Materials and methods

### Materials

Microbiological culturing media used plate count agar (PCA) and potato dextrose agar (PDA) manufactured by Merck (Johannesburg, South Africa). Violet red bile agar (VRBA) was from HiMedia Laboratories Pvt. Ltd (Mumbai, India) whilst the Brilliance *E. coli* / coliform selective medium and xylose lysine deoxycholate agar (XLDA) were procured from Oxoid Ltd (Basingstoke Hampshire, UK).

### Sample collection and preparation

Samples of freshly harvested mopane worms were collected from Gwanda district between March and May 2021 from three randomly identified harvesting camps and three randomly selected harvesters per each harvesting camp. The harvesters were given vinyl reusable hand gloves, plastic disposable aprons, hairnets, clean buckets and polypropylene bags for use during harvesting and handling to reduce contamination. Each harvester harvested 2 kg mopane worms, which were collected and mixed to form a composite sample. The mopane worms were manually degutted, cleaned with potable water and packed in plastic zip lock bags. Within 24 h, the samples were transported to Chinhoyi University of Technology on flaked ice in cooler boxes and stored at -18 °C until further analyses. Prior to analysis, mopane worms were taken out of the freezer and left at room temperature overnight to allow for thawing in a sterile container. Thereafter, the mopane worms were processed (boiling and drying) following the experimental design. After drying, the mopane worms were ground into powder using an AE 099 blender (AE Electrical, Harare, Zimbabwe) that passed through a 600 μm sieve, packed in tightly closed container and stored at 4 °C until further use.

### Experimental design

The traditional mopane worm processing method was optimised using central composite design, response surface methodology (RSM). The impact of the independent variables boiling time (0-20 min) and drying temperature (40-60 °C) were investigated on the protein content and coliform counts (dependent variables) present in the processed mopane worms. The levels of the boiling time and drying temperature were determined from literature (Klunder *et al*., 2012; Lautenschläger *et al*., 2017; Madibela *et al*., 2007; Manditsera *et al*., 2019). An experimental design (13 runs) with uncoded levels which was generated from statistical software Design Expert version 13 (StatEase Inc., Minneapolis, MN, USA) is summarised in Table 1. Randomisation was used in the preparation of the experimental. The systems behaviour was described using a quadratic polynomial model regression (Equation 1).

(1) $$Y=\beta_{0}+{\text{Σ}}^{2}{}_{k=1}\beta_{i}X_{i}+{\text{Σ}}^{2}{}_{k=1}\beta_{ii}X_{i}X_{i}+\varepsilon$$

where Y is the response variable, β_0_ is a constant; β_i_ is the linear and β_ii_ the interactive coefficient; X_i_ is the level of the independent variable and ε is the random error. Maximising protein content and minimising the coliform counts in the processed mopane worms was the optimisation objective. The numerical optimisation at a point maximising the desirability function was determined using Design Expert 13 (StatEase Inc.).

### Protein content determination

Crude protein was determined by the Kjeldahl method (AOAC, 2019) and the nitrogen to protein conversion factor of 4.76 was used for calculations as reported by (Janssen *et al*., 2017).

### Coliform counts determination

Processed mopane worm insect powders were subjected to up to 10^-6^ serial dilution in peptone water. Total coliforms enumeration was done using plate counts on VRBA incubated at 35-37 °C for 24 h.

### Verification and selection for further analysis

The optimum conditions for the processing of mopane worms were compared to the predicted values for verification purposes at 95% confidence interval. The crude fibre, moisture content, ash content, fat content, mineral composition and microbial quality analyses were done on the nine chosen experimental runs after optimising for the protein content and coliform counts.

### Nutrient analysis

Moisture content was determined using a convection oven (Scientific, Johannesburg, South Africa) at 105 °C for 24 h as described by (Nielsen, 2010). Crude fat was gravimetrically determined using the Soxhlet procedure (AOAC, 2019) using petroleum ether as the extracting solvent. After extraction, the petroleum ether was removed using a rotary evaporator (Buchi R-144, Marshall Scientific, Hampton, NH, USA). Ash content was determined by heating the sample in a muffle furnace (Scientific) at 550 °C to constant weight (AOAC, 2019).

Crude fibre was determined following the method previously described by (Anaduaka *et al*., 2021) with minor modifications. Processed mopane worm samples (2.0 g) (F1) were each transferred to a 300 ml conical flask followed by addition of 200 ml of 0.128 M H_2_SO_4_. The mixture was then boiled for 30 min and filtered using a Whatman number 1 filter (Merck, Johannesburg, South Africa). The residue was returned to the conical flask after washing three times with hot water (80 °C). Thereafter, 200 ml of 0.223 M NaOH were added to the mixture and boiled for 30 min followed by filtration using a Whatman number 1 filter. The residue was then washed with 25 ml of 0.128 M H_2_SO_4_, three 50 ml portions of water and 25 ml of ethanol. Thereafter, the residue was transferred to a pre-weighed crucible (F2), ashed at 130 °C for 2 h, cooled in a desiccator and weighed (F3). The samples were then ignited for 30 min at 600 °C, cooled in a desiccator and reweighed (F4). The crude fibre was calculated as loss in weight on ignition as presented in Equation 2:

(2) $$\text{Crude}\;\text{fibre}\;(\text{\%})=\frac{\left(\text{F}3-\text{F}2)-(\text{F}4-\text{F}2\right)}{\text{F}1}\times 100$$

### Mineral profiling

Minerals (Ca, Mg, K, Na, Fe, Zn, Mn) were ashed using nitric acid and perchloric acid following the method previously described by Paul *et al*. (2014) with minor modifications. Briefly, 10 ml of nitric acid and 5 ml of perchloric acid were added to 0.5 g of mopane worm sample in a digestion flask and heated until sample components dissolved. The mixture was cooled and filtered into a 100 ml volumetric flask. The flask was made to the mark with distilled water. Minerals were determined using a Flame Atomic Absorption spectrophotometer (PinAAcle 900F, Perkin Elmer, Midrand, South Africa). For each sample, the analyses were performed in duplicate.

### Microbiological analyses of processed insects

Processed mopane worm insect powders were subjected to up to 10^-6^ serial dilution in peptone water. Cell suspensions were measured using plate counts for enumeration of the following microbial groups: (1) total bacterial count (TBC) on PCA with incubation at 37 °C for 48 h; and (2) yeasts and moulds on PDA, incubated in the dark at 25 °C for 5 days. Enumeration of *E. coli* was done using Brilliance *E. coli* selective medium incubated at 35-37 °C for 24 h. *Salmonella* spp. enumeration was determined using xylose lysine deoxycholate agar (XLDA) incubated at 35-37 °C for 24 h. The selective medias showed *E. coli* and *Salmonella* spp., as purple and red colonies with black centres respectively (Bennett and Lancette, 2001).

### Statistical analysis

Unless otherwise stated, all experiments were carried out in triplicate. Results were analysed and expressed as mean values with standard deviations. Analysis of variance (ANOVA) was used to analyse the significant difference between treatments and Fischer’s Least Significant Differences Test was used to compare means (*P*<0.05 and *P*<0.01).

## 3 Results and discussion

### Optimisation of mopane worm processing method

#### Effect of boiling time and drying temperature on the protein content of the mopane worms

The mopane worm traditional pretreatment methods (boiling time and drying temperature) were evaluated by RSM using central composite design. Table 2 shows the protein content (response) of the mopane worms measured after processing at different boiling times and drying temperatures. The results of the 13 experimental runs from the central composite design showed that the protein content varied with changes in the processing conditions. An increase in the protein content was observed with increasing boiling time. This was in agreement with the study by Megido *et al*. (2018), who reported an increase in the protein content of mealworm (*T. molitor L*.) after boiling for 1 min. In this study, higher protein content (49.4%) was recorded after boiling for 20 min and drying at 40 °C. However, at high drying temperatures irrespective of the boiling time (>0 min) the protein content values were lower. Lautenschläger *et al*. (2017) reported no significant (*P*>0.05) differences in the protein content of raw, boiled and boiled and sun-dried edible caterpillars (*I. epimethea*). The protein content of beetles (*E. Mashona*) was not significantly (*P>*0.05) decreased by boiling for 30 mins. However, increase in the boiling time to 60 min or boiling twice resulted in a significant decrease (*P*<0.05) in protein content of the beetle (Manditsera *et al*., 2019). According to Ursu *et al*. (2016), the small differences in the protein content observed between the various treatments could be due to the loss of small fraction of soluble proteins in the insect exudate.

#### Effect of boiling time and drying temperature on the coliforms in mopane worms

The second dependent variable for the central composite design were the coliforms (which is a hygiene indicator microorganism) in the processed mopane worms. Analysis highlighted the presence of coliforms (Table 2). Coliform counts decreased with an increase in both boiling time and drying temperature. However, boiling had more significant impact on coliform counts. Mopane worms boiled for >10 min had coliform counts within expected limits of less than 10^3^ cfu/g (3 log cfu/g) in foodstuffs that require further cooking (>70 °C) (Lim *et al*., 2012). Hence, the lowest levels of coliform counts (1.20-1.47 log cfu/g) were observed in the mopane worms boiled for 20-24.14 min and dried at 40-60 °C. Thus, the boiled and dried mopane worm samples were safe for human consumption. Mujuru *et al*. (2014), reported a decrease in the coliform counts to acceptable levels in mopane worms that had been boiled and solar dried. Boiling inactivates the insect’s endogenous enzymes that are capable of degrading or spoiling the product (Melgar-Lalanne *et al*., 2019). Exposure of edible insects to heat reduces microbial growth and contamination *(*Wynants *et al*., 2018).

#### Model analysis

The *P*-value for the quadratic model and model terms (A, B) were less than 0.0500 for the protein content and coliforms (Table 3). This indicated that the factors investigated in the study; boiling time and drying temperature had a significant effect on both the coliforms and protein content of the mopane worms. The lack of fit was not significant for both as observed from the *P*-value which was greater than 0.05 (*P*>0.05), implying that both models fitted the experimental data well. Furthermore, the model could adequately describe the relationship between the processing variables and mopane worm protein content as well as the coliform counts. The actual R^2^ values for the models were high (0.99) demonstrating that each of the models could account for 99% of the total variation and has the ability to represent significant relationships amongst the variables investigated. The differences of less than 0.2 implies a reasonable agreement between the predicted R^2^ and adjusted R^2^ hence expressing a good correlation in determining the quality of fit. The experiments had a high probability of representing the whole sample.

**Figure 1:**
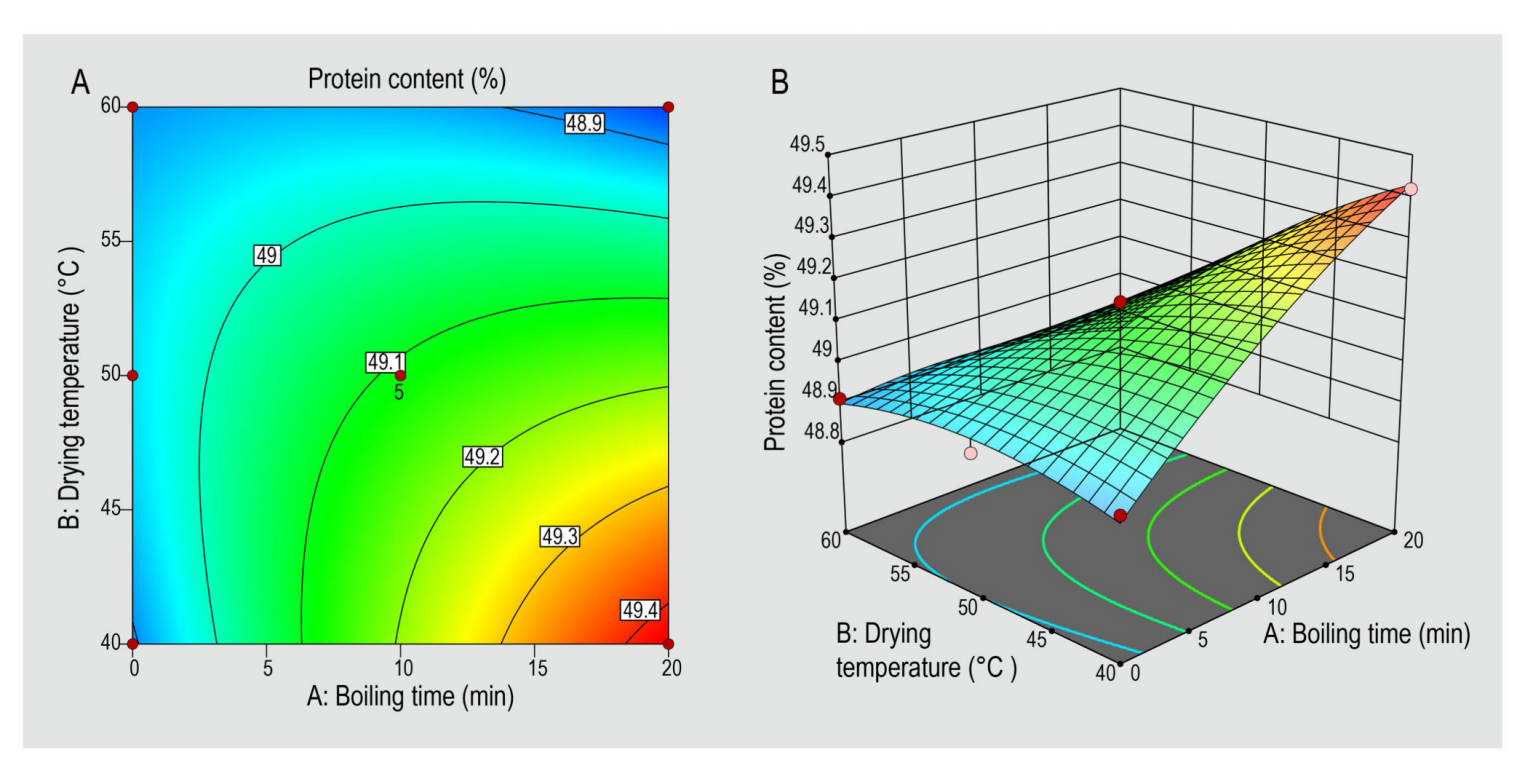
(A) 2-D, and (B) 3-D surface plots showing the interactions effects of the boiling time and drying temperature on the mopane worm protein content.

**Figure 2:**
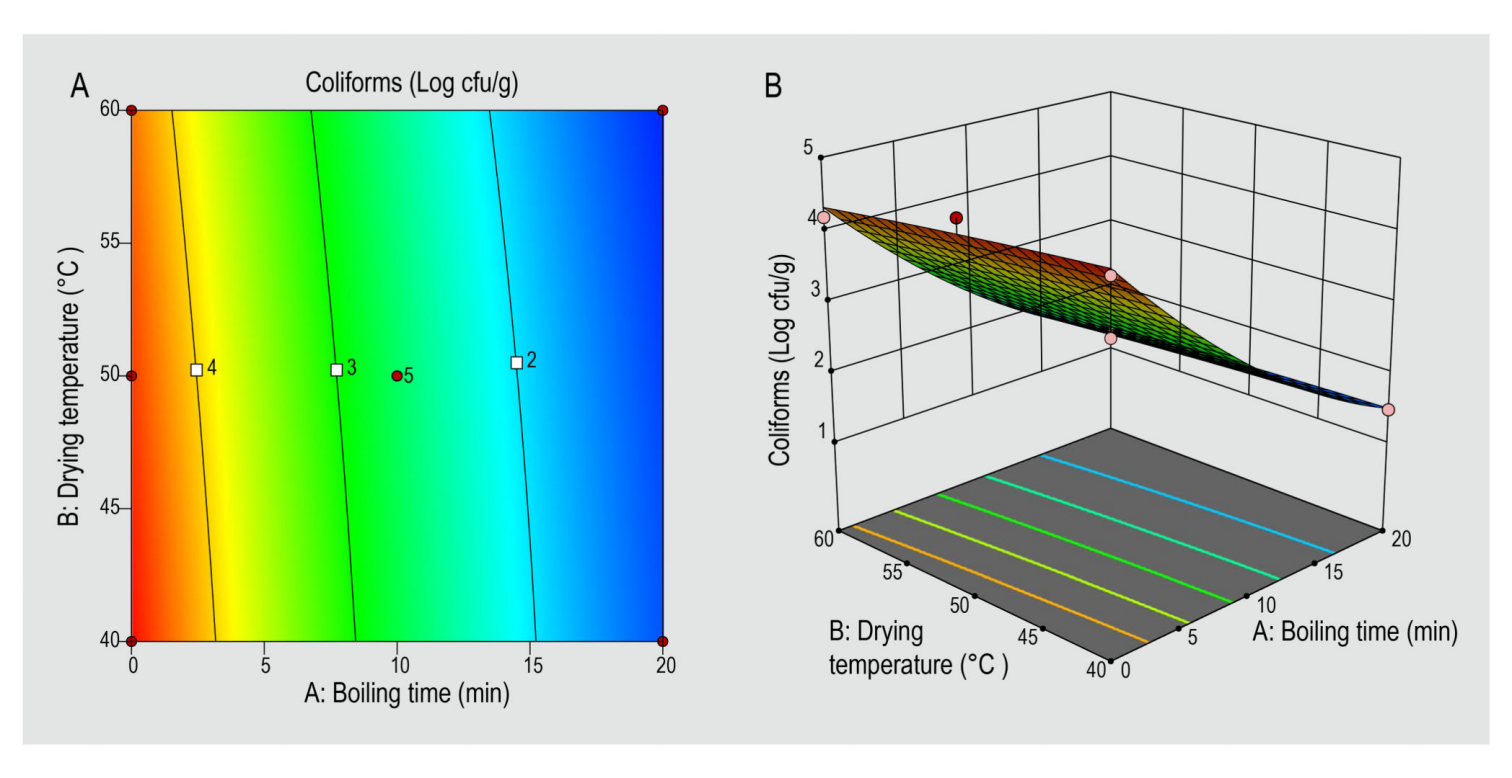
(A) 2-D, and (B) 3-D surface plots showing the interactions effects of the boiling time and drying temperature on the coliforms in mopane worms.

**Figure 3:**
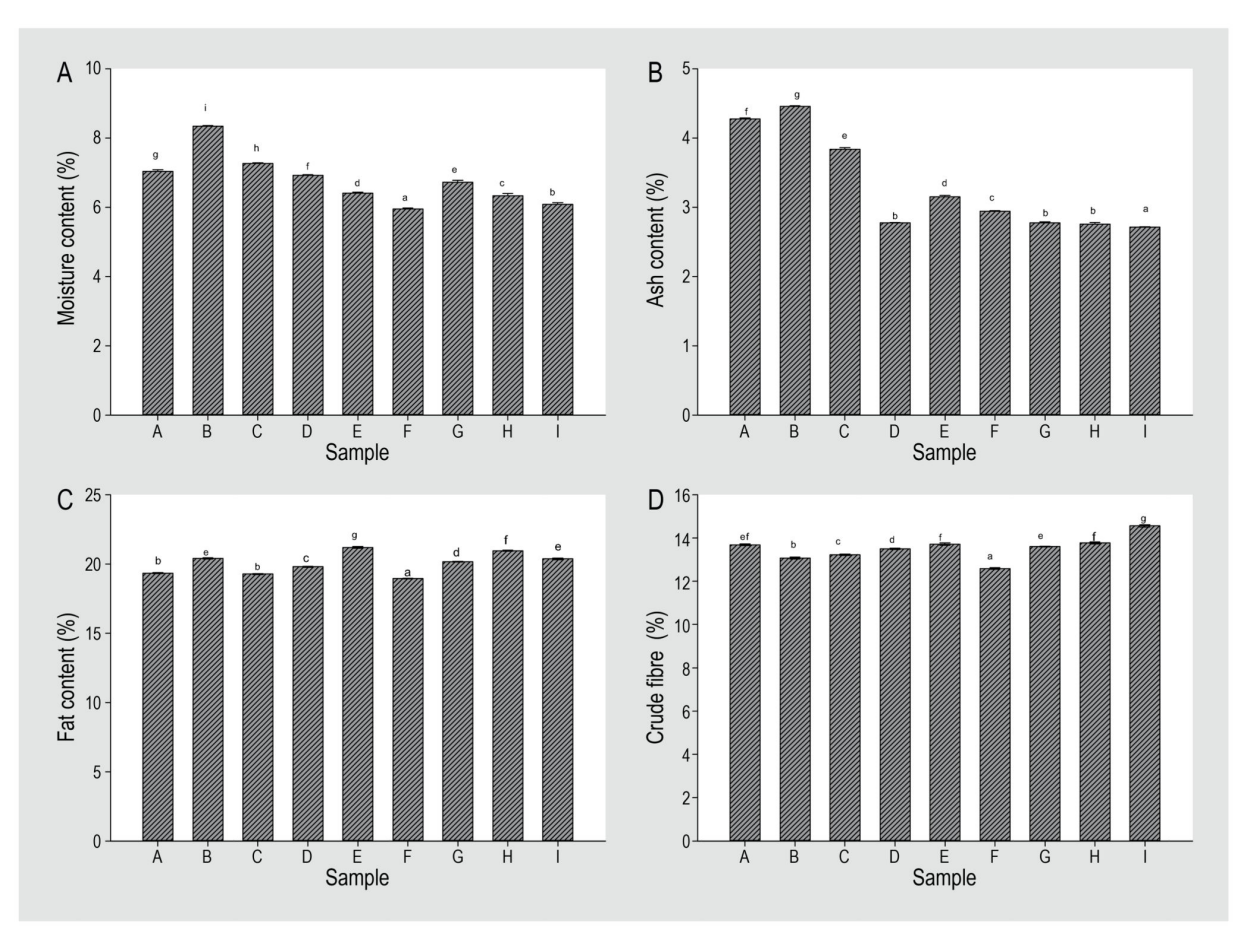
Effect of boiling time and drying temperature on (A) moisture, (B) ash, (C) fat content and (D) crude fibre of mopane worms. (A) 0 min, 40 °C; (B) 0 min, 50 °C; (C) 0 min, 60 °C; (D) 10 min, 35.86 °C; (E) 10 min, 50 °C; (F) 10 min, 64.14 °C; (G) 20 min, 40 °C; (H) 24.14 min, 50 °C; (I) 20 min, 60 °C. Values are means ± SD (n=2). Bars with different small letters differ significantly at *P*<0.05.

The interaction effects of the boiling time and the drying temperature affecting the optimised protein content and coliforms in the mopane worms were also represented in 2-D contour plots (Figure 1A and Figure 2A) and 3-D response surface plot (Figure 1B and Figure 2B). The 2-D contour plots and 3-D response surface plots were deduced to obtain the optimum levels. AB interaction had a significant effect on the mopane worm protein content (Table 3). However, for the other model the AB interaction (Table 3) had no significant effect on the coliforms in the mopane worms. The highest protein content and lowest coliforms were observed at the boiling time of 20 min and drying temperature of 40 °C. The positive effect of the boiling time on the mopane worm protein content is evident through the linear correlation between the two, factor, and response. An increase in the boiling time (0-20 min) is concurrent with the rise in protein content and a decrease in the coliforms in the mopane worms.

### Model validation

The results collected in the experiments were applied to a second-order polynomial regression equations to determine the optimal conditions for the boiling time and drying temperature. The equation coded values (Equation 2 and Equation 3) were used to describe the predicted regression coefficients against calculated values in an equation. Protein content and coliforms in the mopane worms were given as a function of the factors applied in the Equation 2 and 3, respectively.

The polynomial model for the protein content (Y) was regressed by considering the critical terms in Equation 2:

(2) $$\begin{array}{l} \text{Protein}\;\text{content:}\;Y=49.11+0.1224\text{A}-0.1458\text{B}-0.1472\text{AB}\\ -0.0434{\text{A}}^{2}-0.0498{\text{B}}^{2} \end{array}$$

The polynomial model for the coliforms (Y) was regressed by considering the critical terms in Equation 3:

(3) $$\begin{array}{l} \text{Coliforms:}\;Y=2.63-1.5500\text{A}-0.1318\text{B}+0.0525\text{AB}+\\ 0.3533A^{2}-0.0195{\text{B}}^{2} \end{array}$$

The numerical and graphical optimisation technique was used to verify the model adequacy under the selected optimal conditions. The optimisation goal was to maximise the mopane worm protein content. The predicted values were boiling time of 20 min and drying temperature of 40 °C with a desirability of 0.958. The model predicted the maximum protein content and coliform counts of 49.43% and 1.49 log cfu/g respectively, which are not significantly different from the actual levels of 49.42% and 1.47 log cfu/g respectively, confirming the model adequacy. In consideration of the boiling time and drying temperature effects on the mopane worm, further tests (fat, fibre, ash, mineral composition and microbial quality) were therefore done on the selected samples. The main purpose was to ascertain if the optimal processing conditions would produce mopane worms with considerable amounts of fat, fibre and ash, be a good source of minerals like Fe and Zn as well as an improved microbial quality.

#### Effect of boiling time and drying temperature on the nutritional composition of mopane worm

The moisture content (Figure 3A) of the boiled and dried mopane worms were all below 10% suggesting that the samples can be stored at an ambient temperature and be consumed at any time (Vandeweyer *et al*., 2017). Low moisture contents help extend the shelf-life of the mopane worms. Raw and dried samples had higher moisture content as compared to the boiled and dried mopane worms, suggesting that boiling had an impact on the parameter. Moisture content of the mopane worms processed using the optimal conditions was approximately 6.72%. Ash content (Figure 3B) of the processed mopane worms was <5% DW.

Increase in boiling time resulted in a further decrease in the ash content as observed in the higher values in the raw and dried mopane worms. Ash content of the mopane worms processed using the optimal conditions was low (2.78% DW).

Significant differences (*P*<0.01) were observed in the crude fat content (Figure 3C) of the mopane worms after processing at the various parameters of boiling time and drying temperature. Mopane worms processed at the optimal conditions had considerably high crude fat content of approximately 20.16% DW. However, Lautenschläger *et al*. (2017) reported no significant differences in the fat content of raw, boiled and boiled subsequently sun-dried edible caterpillars (*I. epimethea*) which ranged between 8 and 12%. Differences in the crude fat content can be attributed to the combined effect of the processing parameters.

Boiling time and drying temperature had a significant (*P*<0.01) effect on the crude fibre levels of the mopane worms (Figure 3D). The crude fibre levels ranged between 12.5 and 14.6%. Samples boiled for 20 min and dried at 40-60 °C had the highest crude fibre levels. According to Fombong *et al*. (2017), insects contain significant amounts of fibre which is predominantly chitin and complex carbohydrates such as cellulose and lignin that might be present in the insects’ gut. Chitin is a major component of the insect cuticle, which is covalently bound to catechol compounds and sclerotin-like proteins (Hewitson *et al*., 2007).

### Effect of boiling time and drying temperature on the mineral composition of mopane worms

Variations in the boiling time and drying temperature had a significant (*P*<0.01) effect on the content of minerals (Ca, Fe, Zn, Na, Mg, Mn) (Table 4). Boiling time and drying temperature resulted in a significant decrease in the content of Fe and Zn of the mopane worms due to leaching into the cooking water. Madibela *et al*. (2007), also reported a significant reduction in the Zn content of boiled (60 min) mopane worms *(Imbrasia belina)*. The optimally processed mopane worm samples at a boiling time of 20 min and drying temperature of 40 °C still contained considerably a higher concentration of Fe (19.04 mg/100 g) and Zn (17.89 mg/100 g). As iron and zinc deficiency are major public health problem mostly among children in developing countries (FAO *et al*., 2021). Consumption of mopane worms processed under optimal conditions could help in mitigating the deficiencies. The RDI for Fe and Zn in children aged between 4 and 13 years ranges between 8 and 10 mg/d and 5 and 8 mg/d respectively (UNICEF, 2019), suggesting the mopane worm is a good source of Fe and Zn. In comparison to a variety of red meats (1.1-3.3 mg/100 g) (Williams, 2007) the processed mopane worm is a super abundant source of Fe. Da Silva *et al*. 2017), reported that minerals are lost into water during boiling causing the decrease in the mineral content. However, the mineral, boiling duration, the food matrix and the chemical form of the mineral in the food results in variances in the extent of the loss (Manditsera *et al*., 2019).

#### Effect of boiling time and drying temperature on the microbiological quality of mopane worms

The boiling time and drying temperature had a significant (*P*<0.01) effect on the TBC of the mopane worms (Table 5). Mopane worm samples dried at ≤40 °C prior to boiling at 0 and 10 min had TBC counts above the recommended lower limit of log 5.7 cfu/g. Caparros Megido *et al*. (2017), suggested that, the limit for the total bacterial count in edible insects should be at least the same as it is for the fresh minced meat. Total bacterial count limits for fresh minced meat has been specified to a lower limit of 5.7 log cfu/g and an upper limit of 6.7 log cfu/g for five sample units tested from a food batch, with at most two sample units giving values between the lower and upper limits according to the Commission of the European Communities (Megido *et al*., 2018). For all the other processing treatment combinations the TBC levels were less than the lower limit of fresh minced meat.

An increase in the drying temperature resulted in a slight decrease in the TBC. For example, for the dried raw mopane worm samples, an increase in the drying temperature from 40-60 °C resulted in a 6% decrease in the TBC. However, boiling caused a decrease in the TBC of approximately 18-19% suggesting that the boiling time was more effective in reducing the mopane worm microbial load. Furthermore, the results showed that processing reduces the risks involved with the consumption of edible insects (Klunder *et al*., 2012). The results were in agreement with previous studies that reported a decrease in the TBC of mealworms after boiling (Caparros Megido *et al*., 2017; Klunder *et al*., 2012; Megido *et al*., 2018; Vandeweyer *et al*., 2017).

Since microorganisms, were found in the boiled and dried mopane worms, further identification of these organisms was therefore done. Coliforms are usually used as an indicator of unsanitary conditions and poor quality of water and food products (Ramashia *et al*., 2020). A further analysis on the mopane worms was done to determine the levels of *E. coli* and *Salmonella*. Results of *E. coli* highlighted that both boiling time and drying temperature had an impact on their levels (Table 5). Increase in the boiling time from 0-20 min resulted in significant decrease in the *E. coli* levels to 1.15 log cfu/g. Beyond 20 mins, the figure fell below 1.15 log cfu/g. Reports have shown that, the current German and European community threshold for *E. coli* should range from 0-4.0 cfu/g (Grabowski and Klein, 2017b). *Salmonella* spp. were zero in all the mopane worm samples suggesting that the edible insect was safe for human consumption.

According to Ramashia *et al*. (2020), yeast and moulds have the potential to cause spoilage in food products that have low water activity and these include dried edible insects. The highest yeasts and moulds levels were recorded in the raw mopane worm samples which were dried at 40 °C (Table 5). Increase in the drying temperature from 40-60 °C for the raw samples resulted in a significant decrease from 8.01 to 6.88 log cfu/g in the yeast and mould levels. Furthermore, irrespective of the drying temperature, boiling time resulted in a significant decrease in the yeast and mould levels. All the mopane worm samples boiled for ≥10 min had a yeast and mould level <4.5 log cfu/g. In dried edible insects, the microbial limits of yeasts and moulds is 5 log cfu/g (Vandeweyer *et al*., 2018) whilst in animal feed the level was 6 log cfu/g (Stannard, 1997). The lowest yeast and moulds levels were recorded in the mopane worm samples that had been boiled for 20 min and dried at 40-50 °C.

Generally, increase in the boiling time and drying of the mopane worms resulted in a decrease in the microbial loads to levels that are within specification. This should be due to the exposure to heat which reduces the microbial growth and contamination hence capable of decreasing the initial microbial load (Wynants *et al*., 2018). Melgar-Lalanne *et al*. (2019) reported that, boiling inactivates the insect’s endogenous enzymes that are capable of degrading or spoiling the product. Drying reduces the insect’s moisture content and water activity, hence this slows the microbial growth, chemical and enzymatic reactions subsequently increasing the product’s shelf life (Liceaga, 2021).

## 4 Conclusions

Boiling time and drying temperature had significant effect on the nutritional content and microbial safety of the mopane worms. Although, the boiling time and drying temperature interaction had significant effect on the protein content, it had no significant impact on the coliforms. Mopane worms with the highest protein content and minimal coliform levels were processed at optimum conditions of 20 min (boiling time) and 40 °C (drying temperature). These mopane worms had an ash content of 2.78% DW and considerably high contents of crude fat and crude fibre of 20.16% DW and 13.60% DW respectively. The optimal processed mopane worms are a good source of Fe and Zn with concentrations that could contribute significantly to the daily requirements. Furthermore, the mopane worms had improved microbial quality, considered to be safe for human consumption after boiling and drying. Overall, boiling time (20 min) and drying temperature (40 °C) can be used for pretreating harvested mopane worms with improved nutritional composition and microbial quality. Shelf-life studies on pretreated mopane worms packaged in different materials is suggested.

## Figures and Tables

**Table 1:** Process variables used in the central composite design for optimisation of the mopane worm processing method.

Factors	Coded (Xi)				
	-α	-1	0	+1	+α
Boiling time (min)	0	0	10	20	24.14
Drying temperature (°C)	35.86	40	50	60	64.14

**Table 2:** Process variables and responses for optimisation of the mopane worm processing method.

Run	Factor 1	Factor 2	Response 1	Response 2
	A: boiling time Min	B: drying temperature °C	Protein %	Coliforms cfu/g
1	10	50	49.09±0.02	2.66±0.01
2	10	50	49.15±0.02	2.58±0.03
3	0	40	48.91±0.03	4.61±0.08
4	20	60	48.83±0.00	1.26±0.17
5	10	50	49.11±0.01	2.62±0.04
6	10	35.86	49.21±0.03	2.82±0.02
7	20	40	49.42±0.03	1.47±0.09
8	0	50	48.91±0.01	4.74±0.03
9	10	50	49.11±0.02	2.68±0.05
10	10	64.14	48.81±0.02	2.52±0.09
11	10	50	49.11±0.02	2.50±0.13
12	0	60	48.91±0.04	4.19±0.04
13	24.14	50	49.21±0.04	1.20±0.17

**Table 3:** Quadratic model and linear model regression coefficients for the protein content and coliforms of the mopane worm.

Source	Protein content		Coliforms	
	Coefficients	*P*-value	Coefficients	*P*-value
Intercept	+49.1100		+2.6300	
A: boiling time (min)	+0.1217	<0.0001	-1.5500	<0.0001
B: drying temperature	-0.1445	<0.0001	-0.1318	0.0236
AB	-0.1475	<0.0001	+0.0525	0.4437
A^2^	-0.0441	0.0058	+0.3533	0.0005
B^2^	-0.0498	0.0011	-0.0195	0.7011
Model		<0.0001		<0.0001
Lack of fit		0.3107		0.0539
R2		0.99		0.99
Adjusted R2		0.98		0.99
Predicted R2		0.95		0.96

**Table 4:** Effect of boiling time and drying temperature on the mineral composition of mopane worms.^1^

Treatment		Minerals					
Boiling time (min)	Drying temperature (°C)	C (mg/100 g)	Fe (mg/100 g)	Zn (mg/100 g)	Na (mg/100 g)	Mg (mg/100 g)	Mn (mg/100 g)
0	40	69.78±0.01^b^	27.90±0.04^i^	18.92±0.01^i^	3.41±0.01^a^	152.05±0.02^g^	4.30±0.02^f^
0	50	73.24±0.02^e^	18.28±0.01^f^	18.34±0.02^h^	4.44±0.02^d^	153.37±0.01^h^	4.78±0.03^h^
0	60	78.26±0.02^h^	22.04±0.00^h^	17.77±0.01^f^	6.52±0.03^i^	143.47±0.02^f^	3.88±0.02^c^
10	35.86	71.79±0.01^d^	15.77±0.02^e^	17.28±0.03^e^	4.26±0.01^c^	115.27±0.01^c^	3.68±0.02^a^
10	50	62.67±0.02^a^	14.57±0.01^d^	14.77±0.04^c^	3.72±0.02^b^	119.23±0.02^d^	4.56±0.05^g^
10	64.14	70.77±0.00^c^	11.87±0.03^a^	14.53±0.02^a^	4.94±0.01^h^	125.77±0.03^e^	4.07±0.02^e^
20	40	73.25±0.04^f^	19.04±0.01^g^	17.89±0.02^g^	4.46±0.01^f^	113.05±0.01^a^	3.74±0.02^b^
24.14	50	122.23±0.04^i^	14.10±0.04^b^	16.40±0.01^d^	4.52±0.02^g^	166.56±0.01^i^	3.93±0.01^d^
20	60	76.69±0.01^g^	14.28±0.03^c^	14.64±0.02^b^	4.44±0.02e	113.66±0.02^b^	4.90±0.01^i^

1 Values are mean ± SD (n=3); different superscripts indicate significant differences (*P*<0.01) among the different processing treatments.

**Table 5:** Effect of boiling time and drying temperature on the microbiological quality of mopane worms.^1^

Treatment		Microbiological quality			
Boiling time (min)	Drying temperature (°C)	Total bacterial count (log cfu/g)	Yeasts and moulds (log cfu/g)	Escherichia coli (log cfu/g)	Salmonella (log cfu/25 g)
0	40	5.85±0.02^g^	8.01±0.04^g^	3.56±0.12^e^	0
0	50	5.61±0.04^f^	7.38±0.07^f^	3.48±0.03^e^	0
0	60	5.45±0.05^e^	6.88±0.17^e^	3.25±0.03^d^	0
10	35.86	5.81±0.02^g^	4.47±0.02^d^	2.07±0.16^c^	0
10	50	4.93±0.04^d^	4.05±0.08^bc^	1.00±0.00^b^	0
10	64.14	4.60±0.00^b^	3.88±0.09^ab^	1.15±0.21^b^	0
20	40	4.77±0.10^c^	3.82±0.07^a^	1.15±0.21^b^	0
24.14	50	4.54±0.09^ab^	3.90±0.05^ab^	1.00±0.00^a^	0
20	60	4.47±0.00^a^	4.13±0.08^c^	1.00±0.00^b^	0

1 Values are mean ± SD (n=3); different superscripts indicate significant differences (*P*<0.01) among the different processing treatments.

## Data Availability

The data that support the findings of this study are available from the corresponding author, upon reasonable request.
